# Cardiovascular efficacy of sitagliptin in patients with diabetes at high risk of cardiovascular disease: a 12-month follow-up

**DOI:** 10.1186/s12933-016-0371-z

**Published:** 2016-03-31

**Authors:** Takashi Nakamura, Yoshitaka Iwanaga, Yuki Miyaji, Ryuji Nohara, Takao Ishimura, Shunichi Miyazaki, on behalf of the Sitagliptin Registry Kinki Cardiologists’ Study (SIRKAS) Investigators

**Affiliations:** Division of Cardiology, Department of Internal Medicine, Faculty of Medicine, Kinki University, 377-2 Ohno-Higashi, Osakasayama, 589-8511 Japan; Hirakata Kousai Hospital, Hirakata, Japan; Ishimura Clinic, Osaka, Japan

**Keywords:** Blood pressure, Cardiovascular risk, Type 2 diabetes, Sitagliptin, Urine albumin

## Abstract

**Background:**

Gliptins should have beneficial effects beyond glycemic control, potentially on the pathophysiology of cardiovascular (CV) diseases, with some basic studies demonstrating this possibility. However, we are yet to answer whether there are any direct CV effects in the clinical setting. We aimed to examine the beneficial effects of sitagliptin in Japanese patients with diabetes and high CV risk for 12 months.

**Methods:**

This was a prospective, multicenter, observational study of 205 patients with type 2 diabetes. All participants had more than one major CV risk factor and were treated with sitagliptin for 12 months. At 3 or 12 months, we examined the effects of treatment on glycemic control, CV function (by electrocardiography, echocardiography, and reactive hyperemia-peripheral arterial tonometry), and CV biomarkers.

**Results:**

Patients were predominantly elderly (68.8 ± 9.9 years) and male (71.5 %) and typically had more than three CV risk factors (79.2 %). Treatment with sitagliptin significantly reduced the hemoglobin A1c (HbA1c) level from 7.09 % ± 0.81 % at baseline to 6.67 % ± 0.69 % at 3 months and 6.68 % ± 0.73 % at 12 months (both P < 0.001). The reduction in HbA1c was also in tandem with the decrease in the level of high-sensitive C-reactive protein throughout the study. Independent of the change in HbA1c, sitagliptin reduced systolic (−7.0 ± 18.9 mmHg) and diastolic blood pressure (−5.1 ± 11.7 mmHg) at 12 months, and this was associated with a decrease in urinary albumin. In contrast, there were no beneficial effects on cardiac and endothelial function or on the levels of serum B-type natriuretic peptide, high-sensitive troponin T, and urinary 8-hydroxy-2′-deoxyguanosine.

**Conclusions:**

In Japanese patients with diabetes and multiple CV risk factors, sitagliptin showed a decrease in blood pressure associated with an improvement in albuminuria in addition to glycemic control.

*Trial registration:* UMIN000005663

**Electronic supplementary material:**

The online version of this article (doi:10.1186/s12933-016-0371-z) contains supplementary material, which is available to authorized users.

## Background

Type 2 diabetes is strongly associated with coronary atherosclerosis and vascular complications, both of which are responsible for worse morbidity and mortality [1]. Although the pathogenesis of atherosclerosis and associated complications remains unclear [2], inflammatory processes may be a critical step, constituting a biological link between diabetes and vascular disease [3]. Optimal management of type 2 diabetes includes pharmacological interventions and life-style modification, including diet, exercise, and weight loss. To reduce the risk of cardiovascular (CV) events, not only is pharmacological therapy necessary for lowering blood sugar levels but antihypertensive, antihyperlipidemic, and antiplatelet therapies are also needed [4]. In particular, integrated behavior modification and targeted polypharmacy are especially beneficial for patients with diabetes who are at high risk of CV disease (CVD) [5].

Sitagliptin, a dipeptidyl peptidase-4 (DPP-4) inhibitor (gliptin), has been shown to increase plasma incretin hormone levels, including glucagon-like peptide-1 (GLP-1) and glucose-dependent insulinotropic polypeptide (GIP). Increases in these peptides appear to be responsible for the principle mechanism of action of sitagliptin; as the levels of GLP-1 increase, glucose concentrations decrease [6]. Sitagliptin is known to be effective and safe as both monotherapy and combination therapy in patients with diabetes. Recently, the Trial Evaluating Cardiovascular Outcomes with Sitagliptin (TECOS) assessed composite CV endpoint and hospitalization rates for heart failure in patients with type 2 diabetes and established CVD, and demonstrated that sitagliptin was safe [7]. However, we still do not know the various long-term influences of incretin-based therapies, such as sitagliptin, on CV risk factors and disease, including the effects on morbidity and mortality.

DPP-4 is a widely expressed glycoprotein that exists either as a transmembrane protein or in a soluble form in plasma, and has many targets other than GLP-1 and GIP. In addition, the GLP-1 receptor is variously expressed, being located not only in pancreatic cells but also in the lungs, kidneys, intestines, peripheral and central nervous systems, and the CV system. Based on this, it has been suggested that gliptins should have beneficial effects beyond glycemic control, potentially on the pathophysiology of CVD, with some basic studies demonstrating this possibility [8]. However, these effects remain unclear in the clinical setting [9], and the factors predictive of a blood glucose-lowering effect remain unknown in patients with diabetes who are at high risk of CVD. Equally, we are yet to answer whether there are any direct CV effects in the medium term.

We performed this prospective study to determine whether sitagliptin therapy for 12 months had any beneficial effects on glycemic control and CV function in Japanese patients with diabetes at high risk of CVD.

## Methods

### Study design

This was a prospective, multicenter, observational study. Eligible patients with diabetes at high risk of CVD were treated with sitagliptin for 12 months from August 2011 to September 2012, at nine hospitals and clinics in Japan. The patients received sitagliptin at dosages of 25, 50, or 100 mg/day at the discretion of the attending physician, and biochemical markers, urinalysis, electrocardiography (ECG), and echocardiography (UCG) were assessed before, and at 3 and 12 months after treatment. In a sub-study at the Department of Internal Medicine, Kinki University Faculty of Medicine and the Tazuke Kofukai Medical Research Institute, Kitano Hospital, we also performed reactive hyperemia-peripheral arterial tonometry (RH-PAT) before and at 3 months after starting sitagliptin and measured CV biomarkers before and at 3 and 12 months after treatment.

The study protocol was approved by the Ethics Committee of Kinki University Faculty of Medicine and Osaka Foundation for the Prevention of Cancer and Cardiovascular Disease. Written informed consent was obtained from all patients. The trial was registered at http://www.umin.ac.jp under UMIN000005663.

### Selection criteria

The inclusion criteria were as follows: (1) age ≥20 years; (2) type 2 diabetes with a hemoglobin A1c (HbA1c) ≥6.2 %, a fasting glucose ≥110 mg/dL, or a postprandial glucose ≥140 mg/dL despite appropriate diet and exercise, oral antihyperglycemic agents, or insulin therapy; and (3) at least one CV risk from among hypertension, dyslipidemia, previous CV event, advanced age (≥65 years), current smoking, obesity (body mass index ≥25), and hyperuricemia. Patients were excluded if they met the following criteria: (1) history of diabetic coma and ketoacidosis within 6 months before the study, (2) serious trauma or infectious disease, (3) currently nursing or pregnant women, (4) dialysis or severe renal insufficiency (creatinine clearance <30 mL/min), and (5) known hypersensitivity to any component of the study medications.

### Study endpoints

The primary endpoint was the change in HbA1c after 3 and 12 months of sitagliptin therapy. The major secondary endpoints were changes in body weight, blood pressure (BP), biochemical measures, urinary measures, ECG parameters, and UCG parameters.

### Ambulatory BP measurement

BP was measured during office visits at baseline and after 3 and 12 months of treatment. Cuffs were used with the bladder encircling at least 80 % of the mid-arm circumference and placed directly on the skin. All BP measurements were taken after 5 min of rest with patients sitting and with the arm resting on a table at the level of the heart.

### Measurement of biochemical and urine parameters

The following parameters were measured at baseline and after 3 and 12 months of treatment: standard urinalysis, urinary albumin, complete blood count, serum electrolytes, liver function, renal function, lipid profile, immunoreactive insulin, and HbA1c. In the sub-study, we also measured the following before and at 3 and 12 months after treatment: urinary 8-hydroxy-2′-deoxyguanosine (8-OHdG) as a marker of oxidative stress, high-sensitive C-reactive protein (hs-CRP) as an inflammatory marker, and both high-sensitive troponin T (hs-TnT) and B-type natriuretic peptide (BNP) as cardiac markers. The estimated glomerular filtration rate (eGFR) was calculated using the equation specific to the Japanese population: eGFR = 194 × (serum creatinine)^−1.094^ × (age)^−0.287^ (× 0.739 if female) [10]. Albuminuria was determined by the urinary albumin-to-creatinine ratio (UACR). Microalbuminuria was defined as a UACR of 30–300 mg/g creatinine, and overt albuminuria as a UACR of 300–3000 mg/g creatinine. The homeostasis model assessment-insulin resistance (HOMA-IR) value was calculated using the following formula: HOMA-IR = [fasting insulin (μIU/ml) × fasting glucose (mg/dl)]/405.

### ECG and UCG examination

ECG and UCG were performed at baseline and after 12 months of treatment. The ECG parameters were examined by automatic analysis [ECG-1450 (Nihon Kohden, Tokyo, Japan)]. Heart rate (HR), rhythm, PR interval, QRS interval, QT/corrected QT (QTc) interval, and the SV1 + RV5 value were obtained [11]. In the UCG analysis, the chamber dimensions, left ventricular (LV) ejection fraction (EF), and LV mass index were assessed from M-mode images using the American Society of Echocardiography-recommended formula [12]. For patients in sinus rhythm, the pulsed Doppler transmitral flow velocity was recorded to measure the ratio of peak mitral E-wave velocity to peak mitral A-wave velocity (E/A ratio) and the deceleration time of the mitral E-wave velocity (Dct). The peak of myocardial early diastolic velocity (e′) in the septal annulus was also measured by tissue Doppler imaging, and the ratio of peak mitral E-wave velocity to e′ velocity (E/e′ ratio) was calculated for the assessment of LV relaxation.

### Assessment of endothelial function

In the sub-study, we assessed peripheral endothelial function by RH-PAT using an EndoPAT2000 (Itamar Medical, Caesarea, Israel), as described previously [13]. RH-PAT was performed at baseline and after 3 months of treatment to calculate the reactive hyperemia index (RHI), which reflects the extent of reactive hyperemia, and thereby, endothelial function. It was calculated as the ratio of the average amplitude of the PAT signal over 1 min starting at 1.5 min after cuff deflation (control arm, A; occluded arm, C) divided by the average amplitude of the PAT signal of a 2.5-min time period before cuff inflation (baseline) (control arm, B; occluded arm, D), as follows: RHI = (C/D)/(A/B).

### Statistical analysis

Categorical variables were compared using the χ^2^ test for proportions and the unpaired *t* test or analysis of variance (ANOVA) for continuous variables, as appropriate. The linearity of the relationship between two variables was assessed by linear regression analysis, and Pearson’s correlation coefficient was calculated. Differences between the reduction or increase in each marker and the change in HbA1c were compared using Student’s *t* tests. Further multiple logistic regression analysis was performed to define independent variables that might predict the change in HbA1c. Overall differences between before and after sitagliptin administration were determined using repeated measures ANOVA. All P values <0.05 were considered statistically significant. Results are expressed as the mean ± standard deviation (SD). All analyses were performed using JMP version 10.0.

## Results

### Baseline clinical characteristics

Of the 211 patients enrolled, 6 patients were excluded from the analysis because they discontinued sitagliptin after 3 months, citing dizziness (2 patients), mild hypoglycemia (1 patient), headache (1 patient), or general malaise (1 patient). However, no severe adverse events, including severe hypoglycemia, were reported. One patient withdrew consent. The baseline characteristics of the 205 participants are shown in Table 1. Most were male (71.5 %) and aged over 65 years (73.4 %), with mean systolic and diastolic BPs of 133.6 ± 19.2 and 74.8 ± 11.6 mmHg, respectively. Three or more CV risk factors were present in 79.2 %, with hypertension being most common. However, 42.0 % had some medical history of CVD, including coronary heart disease. Over the study period, the mean sitagliptin dosage was 52.0 ± 12.7 mg/day. About half of the patients (52.6 %) received sitagliptin monotherapy and the remainder received combination therapy, with the most common co-administered drugs being sulfonylureas (21.3 %) followed by α-glucosidase inhibitors (17.9 %) and thiazolidinediones (14.5 %).

**Table 1:** Baseline clinical characteristics

	N = 205	
Age (years)	68.8 ± 9.9 (37–88)	
Male (%)	71.5	
BMI (kg/m^2^)	25.7 ± 4.1	
Blood pressure (systolic/diastolic) (mmHg)	133.6 ± 19.2/74.8 ± 11.6	
Cardiovascular risk factors ≥3 (%)	79.2	
Hypertension	87.0	
Dyslipidemia	65.2	
Cardiovascular disease	42.0	
Coronary artery disease	39.6	
ASO	2.4	
Cerebral infarction	2.9	
Senior (≥65 years)	73.4	
Current smoking	16.6	
Obesity (BMI ≥25)	52.9	
Hyperuricemia (≥7.0 mg/dL)	19.9	
Combination of oral hypoglycemic drugs (%)	Pre-	Post-
None	52.6	56.3
Sulfonylurea	21.3	19.4
α-Glucosidase inhibitor	17.9	16.5
Thiazolidinedione	14.5	12.6
Biguanide	10.6	10.6
Glinide	6.3	2.4
ACEI/ARB	63.6	
Calcium channel blocker	41.5	
Statin	58.3	

Values are the mean ± SD or %

*ACEI* angiotensin-converting enzyme inhibitor, *ARB* angiotensin receptor blocker, *ASO* arteriosclerosis obliterans, *BMI* body mass index

### Blood glucose and urinary changes (Table 2)

At baseline, HbA1c and fasting blood glucose were 7.09 ± 0.81 % and 139.8 ± 33.0 mg/dL, respectively, and both parameters significantly reduced by 3 and 12 months (both P < 0.001). However, there were no changes in insulin, HOMA-IR, low-density lipoprotein cholesterol, triglyceride, or the uric acid level. Only high-density lipoprotein (HDL) cholesterol level reduced significantly. In addition, the eGFR decreased significantly during the study period, but changes in the UACR were not significant.

**Table 2:** Serial changes in clinical and laboratory parameters

	Baseline	3 months	12 months	P value
Body weight (kg)	67.3 ± 14.0	67.5 ± 14.1	67.9 ± 14.1	0.042
HbA1c (%)	7.09 ± 0.81	6.68 ± 0.69	6.69 ± 0.72	<0.001
FPG (mg/dL)	139.8 ± 33.0	130.2 ± 27.2	131.7 ± 29.0	<0.001
IRI (IU)	11.3 ± 10.1	12.4 ± 14.3	11.8 ± 11.1	0.720
HOMA-R	3.9 ± 3.8	3.9 ± 3.8	3.9 ± 3.8	0.914
Insulin resistance (HOMA-R ≥2.5) (%)	54.7	49.4	51.4	
LDL-C (mg/dL)	100.0 ± 24.2	101.4 ± 26.8	99.5 ± 28.6	0.509
HDL-C (mg/dL)	52.4 ± 14.1	51.8 ± 13.4	50.0 ± 12.0	0.036
Triglyceride (mg/dL)	136.1 ± 77.6	131.5 ± 71.8	133.6 ± 68.4	0.308
Uric acid (mg/dL)	5.9 ± 1.2	6.0 ± 1.4	5.8 ± 1.3	0.054
Cr (mg/dL)	0.86 ± 0.25	0.88 ± 0.27	0.88 ± 0.30	<0.001
eGFR (mL/min/1.73 m^2^)	67.7 ± 18.3	66.3 ± 18.3	66.6 ± 18.6	0.009
eGFR <60 (%)	35.9	40.3	35.3	
UACR (mg/g Cr)	79.8 ± 211.4	119.3 ± 483.2	100.5 ± 239.9	0.693
Micro- or overt-albuminuria (%)	40.2	46.3	39.5	

Values are the mean ± SD or %

*Cr* Creatinine, *eGFR* estimated glomerular filtration rate, *FPG* fasting plasma glucose, *HbA1c* glycated hemoglobin, *HDL*-*C* high-density lipoprotein cholesterol, *HOMA*-*R* homeostasis model assessment-insulin resistance, *IRI* immunoreactive insulin, *LDL*-*C* low-density lipoprotein cholesterol, *UACR* albumin-to-creatinine ratio

**Fig. 1:**
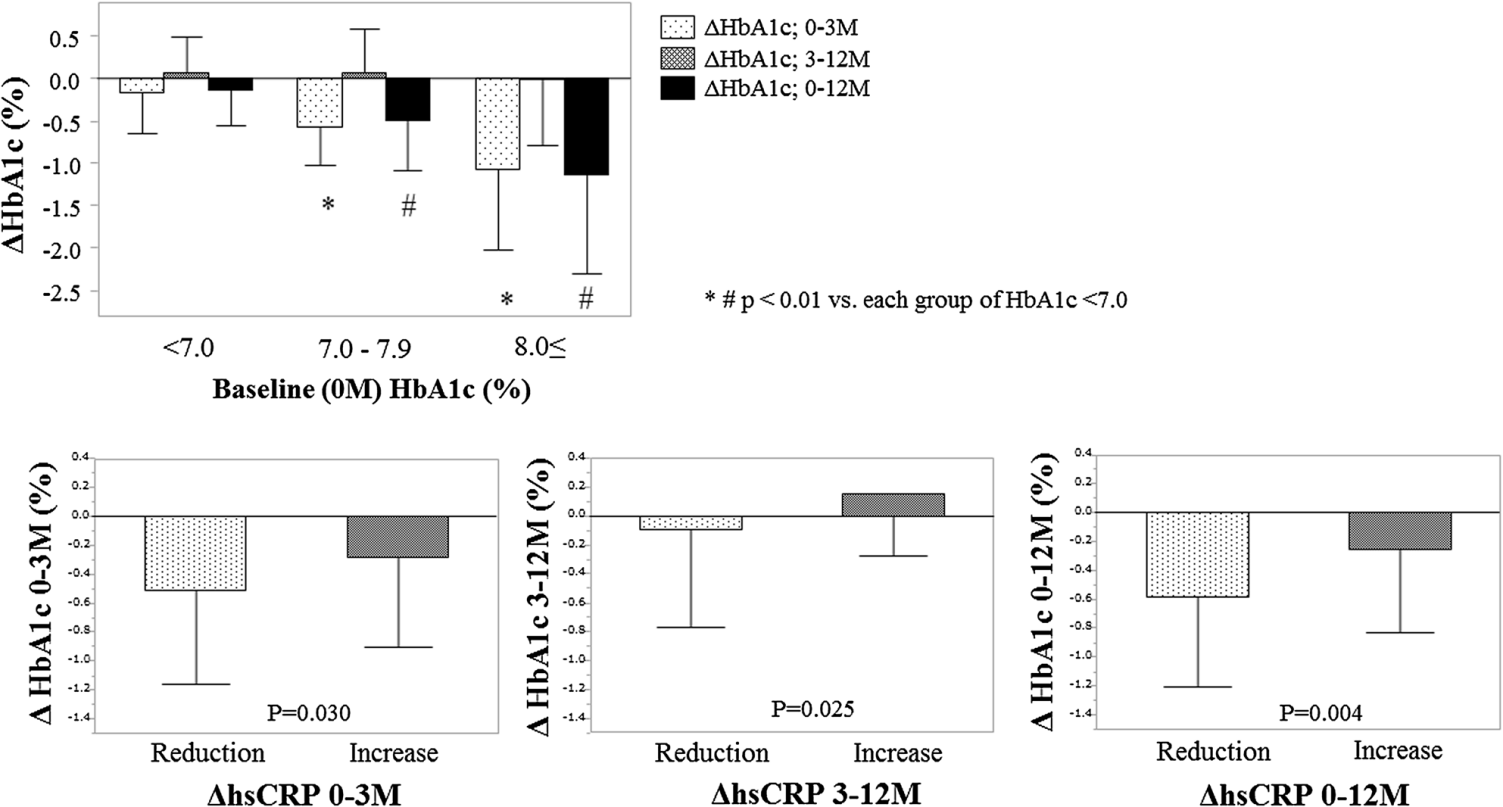
HbA1c change (ΔHbA1c) by baseline HbA1c categories and the relationship with hsCRP change (ΔhsCRP). *HbA1c* hemoglobin A1c, *hs-CRP* high-sensitive c-reactive protein, *M* month. The values are mean ± SD

The changes in HbA1c (ΔHbA1c) at 3 and 12 months were significantly associated with the baseline HbA1c level (Fig. 1). When the predictive factors for ΔHbA1c were examined by multiple regression analysis (see Additional file 1: Table S1), only baseline HbA1c remained a significant factor for ΔHbA1c at 3 months (β = −0.608, P < 0.001). However, at 12 months, significant determinants of ΔHbA1c were age (β = −0.207, P = 0.005), change in body weight (β = 0.148, P = 0.042), and baseline HbA1c level (β = −0.435, P < 0.001).

### Pro-inflammatory biomarkers, CV biomarkers, and CV function

We studied pro-inflammatory biomarkers, CV biomarkers, and CV function in a subpopulation of 127 patients. The hs-CRP, BNP, and urinary 8-OHdG levels remained unchanged after 3 and 12 months of sitagliptin therapy (see Additional file 2: Table S2), but the hs-TnT level significantly increased by 12 months. When the changes in BNP, hs-TnT, and urinary 8-OHdG were divided into groups with either a reduction or an increase, we found no association with ΔHbA1c (all P = n.s.). In contrast, the changes in hs-CRP were significantly associated with changes in HbA1c at both 3 and 12 months (P = 0.030 and 0.004, respectively), suggesting that the effect of sitagliptin on HbA1c levels is blunted in patients with increased hs-CRP levels (Fig. 1).

Among the ECG parameters, HR changed during the 12 months of sitagliptin therapy from 61.2 ± 10.5 beats per minute (bpm) at baseline to 65.0 ± 11.9 bpm at 12 months (P < 0.01). However, although the QT interval shortened significantly (427.9 ± 34.0 ms at baseline to 417.9 ± 34.5 ms at 12 months, P < 0.001), the QTc interval did not change (428.1 ± 22.6 ms at baseline to 425.2 ± 21.9 ms at 12 months, P = 0.093). Other parameters did not change, including the rhythm, PR interval, QRS interval, and SV1 + RV5 value.

In the UCG examination, the LV mass index significantly decreased during the 12 months of treatment, from 109.9 ± 33.5 g/m^2^ at baseline to 105.1 ± 31.9 g/m^2^ at 12 months (P = 0.027). However, the other parameters, including LV dimension, EF, E/A, Dct, e′, and E/e′, showed no changes between baseline and 12 months (Table 3). In the RH-PAT analysis, the RHI did not change by 3 months of sitagliptin treatment (1.92 ± 0.48 at baseline to 1.87 ± 0.80 at 3 months, P = 0.648), and the proportion of patients with an RHI ≤ 1.67 did not change significantly from baseline to 12 months (51.6–42.4 %).

**Table 3:** Changes in electrocardiography and echocardiography parameters

	Baseline	12 months	P value
Electrocardiography parameters			
HR (bpm)	61.2 ± 10.5	65.0 ± 11.9	<0.0001
Sinus rhythm (%)	90.1	89.0	
PR interval (ms)	179.5 ± 32.2	180.5 ± 31.4	0.5784
QRS interval (ms)	98.0 ± 19.1	97.9 ± 18.2	0.7720
SV1 + RV5 voltage (mm)	2.7 ± 0.8	2.6 ± 0.7	0.1288
QT interval (ms)	427.9 ± 34.0	417.9 ± 34.5	0.0004
QTc interval (ms)	428.1 ± 22.6	425.2 ± 21.9	0.0933
Echocardiography parameters			
LV Dd (mm)	49.9 ± 6.3	49.5 ± 6.3	0.0803
LV Ds (mm)	33.2 ± 8.2	32.7 ± 7.9	0.0720
IVST (mm)	8.9 ± 1.9	8.9 ± 1.8	0.7381
PWT (mm)	9.3 ± 6.0	9.3 ± 6.0	0.6180
EF (%)	59.7 ± 12.3	60.0 ± 11.8	0.4006
LV mass index (g/m^2^)	109.9 ± 33.5	105.1 ± 31.9	0.0270
E-wave velocity (m/s)	68.2 ± 19.5	68.4 ± 20.0	0.7217
A-wave velocity (m/s)	78.6 ± 21.8	80.7 ± 20.8	0.3428
Dct (ms)	216.9 ± 58.5	223.9 ± 54.8	0.3279
E/A ratio	0.91 ± 0.45	0.85 ± 0.31	0.2005
e′ (cm/s)	6.08 ± 2.06	5.87 ± 1.59	0.0938
E/e′ ratio	12.11 ± 5.20	12.34 ± 5.22	0.4858

Values are the mean ± SD or %

*Dct* deceleration time, *Dd* dimension of diastole, *Ds* dimension of systole, *EF* ejection fraction, *HR* heart rate, *IVST* interventricular septal thickness, *LV* left ventricular *PWT* posterior wall thickness

### Changes in BP and renal function

**Fig. 2:**
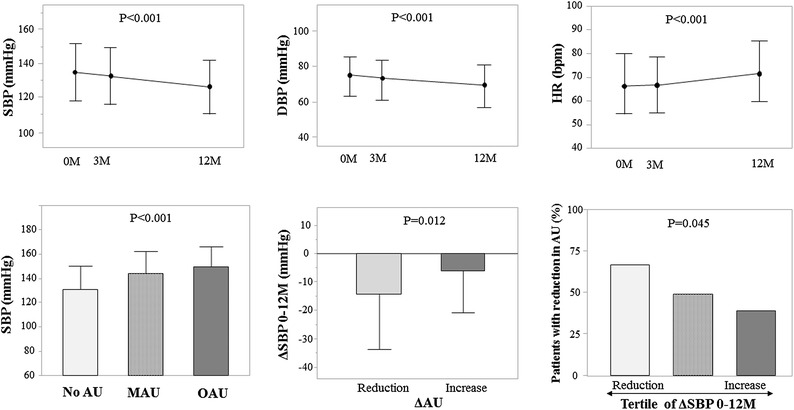
Changes in blood pressure and heart rate, and the relationship with the change in albuminuria. *AU* albuminuria, *DBP* diastolic blood pressure, *MAU* microalbuminuria, *OAU* overt albuminuria, *HR* heart rate, *M* month, *SBP* systolic blood pressure. The values are mean ± SD or %

The results are summarized in Fig. 2. During treatment, systolic BP (SBP) and diastolic BP (DBP) were significantly reduced from baseline (SBP, 133.6 ± 19.2 mmHg; DBP, 74.8 ± 11.6 mmHg) to 12 months (SBP, 127.5 ± 16.4 mmHg; DBP, 70.1 ± 12.4 mmHg). The proportion of patients with a BP ≥140/90 mmHg significantly decreased from baseline to 12 months (33.7–25.3 %). Of the 94 patients who used an antihypertensive agent at baseline and completed the 12-month assessment period, 15 (16.0 %) changed the drug or the dosage during the period. When these patients were excluded, significant changes were still observed in SBP and DBP at 12 months (−11.7 ± 18.6 and −7.3 ± 11.6 mmHg for ΔSBP and ΔDBP, respectively). However, ΔHbA1c by 12 months was associated with neither ΔSBP (r = 0.046, P = 0.318) nor ΔDBP (r = 0.099, P = 0.199). In addition, HR increased from 66.9 ± 13.0 bpm at baseline to 71.3 ± 12.9 bpm at 12 months. However, ΔHR was not associated with ΔSBP (r = 0.046, P = 0.563) or ΔDBP (r = 0.036, P = 0.644) at 12 months (see Additional file 3: Figure S1).

Albuminuria at baseline was positively associated with SBP at baseline. Moreover, the changes in albuminuria (ΔAU) and in systolic BP (ΔSBP) over 12 months were significantly associated; patients with a reduction in albuminuria (n = 63) showed a greater reduction of SBP over the same period than those with an increase in albuminuria (n = 57) (ΔSBP; −14.3 ± 19.3 vs. −6.2 ± 14.6 mmHg, respectively). The tertile of ΔSBP (n = 40 each) was negatively correlated with a decrease in albuminuria (P = 0.045). In contrast, although ΔSBP or ΔDBP was significantly associated with ΔeGFR (r = 0.193, P = 0.012 and r = 0.256, P < 0.001, respectively) (see Additional file 3: Figure S1), ΔAU and ΔeGFR were not associated after 12 months of treatment (r = 0.079, P = 0.375). Finally, ΔAU was not associated with ΔHbA1c (r = 0.046, P = 0.604).

## Discussion

Gliptins can exert favorable cardiovascular effects in experimental models. These changes, as an almost general rule, include improved endothelial function, reduction of inflammatory markers, oxidative stress, ischemia/reperfusion injury and atherogenesis. In addition, increased adiponectin levels and modest decreases in lipidemia and blood pressure were reported [8]. Unfortunately, the favorable DPP4 inhibition features reported in experimental studies are not necessarily reflected in clinical studies [7] and a definite relationship between gliptins treatment and improved CV outcomes remains uncertain and requires further clarification [9, 14]. This could be due to the fact that in addition to the activation of incretin hormones, gliptins are multi-target compounds and may therefore affect various additional substrates, leading to undesirable effects [15].

### Effect of sitagliptin on CV function and biomarkers

Interestingly, the reduction in hs-CRP levels was independently associated with the reduction in HbA1c levels. A recent cross-over study showed that 6 weeks of treatment with sitagliptin significantly reduced the plasma levels of CRP and that there was a significant inverse correlation between changes in GLP-1 and CRP levels following sitagliptin therapy (r = 0.41, P = 0.01) [16]. In the present study, sitagliptin did not significantly decrease hs-CRP levels (P = 0.0994), and the baseline hs-CRP level was not associated with ΔHbA1c (data not shown); therefore, the causal relationship between these factors remains unknown. However, these data suggest that there is a relationship between improvements in glucose–insulin homeostasis and low-grade inflammation that is somehow mediated by sitagliptin therapy in diabetic subjects with high CV risk.

Prolongation of the QT interval is an important risk factor for ventricular arrhythmia and sudden death. In the present study, the QT interval was significantly shortened from baseline to 12 months (P < 0.001), but there was no significant difference in the QT interval after correction for the change in HR (QTc interval) (P = 0.093). The basic research by Lee et al. showed that sitagliptin treatment could reverse calcium regulation in cardiomyocytes, which might contribute to its shortening effects on the QT interval in hypertensive animals [17]. In other research, sitagliptin was not associated with prolongation of either the QT or QTc interval at clinically relevant concentrations in healthy individuals [18]. Although the shortening of the QT interval in this study was mainly associated with the increased HR, the QTc interval still showed a trend toward shortening after sitagliptin therapy (P = 0.093). Thus, it is possible that sitagliptin therapy might lower the risks of ventricular arrhythmias and sudden death due to QT interval change, but further studies are clearly necessary to clarify whether benefits truly exist, particularly in patients with diabetes and comorbid CVD.

GLP-1 receptors are located in cardiac myocytes, coronary vascular endothelial cells, and smooth muscle cells; and, mainly in basic studies, GLP-1 has been reported to improve cardiac function and provide cardioprotective effects [9]. Nikolaidis et al. reported that GLP-1 infusion in patients with acute myocardial infarction improved LV function compared with controls receiving standard care [19]. However, in a randomized study of 80 diabetic patients with LV diastolic dysfunction, Oe et al. reported that sitagliptin for 6 months did not improve diastolic function [20]. In the present study, in which we used patients with similar background characteristics, there was no significant change in BNP levels at 12 months, which is a reasonably sensitive maker for the cardiac dysfunction and development of HF. This was the same for the EF and septal e′ or E/e′, which reflect diastolic function or end-diastolic pressure, as the diagnostic and therapeutic index of HF. Endothelial dysfunction may also result from hyperglycemia and atherosclerosis, and some studies have suggested that sitagliptin can improve endothelial dysfunction in patients with type 2 diabetes and that gliptins may protect endothelial function through a GLP-1-dependent mechanism [21, 22]. In contrast, Aoyagi et al. recently reported that gliptins attenuated endothelial function evaluated by flow-mediated vasodilatation [23]. Also, sitagliptin has been shown to increase adiponectin levels [24, 25], which could be associated with the anti-atherosclerotic effects. Although the measurement was not performed in the present study, sitagliptin treatment did not reduce markers of oxidative stress (urinary 8-OHdG) or inflammation (hs-CRP) but decreased HDL cholesterol levels. They did not affect endothelial function (RHI assessment) favorably. Thus, controversy remains around these aspects of treatment, and further investigation is needed.

### Effect of sitagliptin on BP and albuminuria

We showed that sitagliptin reduced SBP and DBP independent of ΔHbA1c and that this was associated with a reduction in the level of albuminuria. It might also have been associated with a reduction in the LV mass index. Relatively few studies have examined the effects of DPP-4 inhibitors on BP in patients with diabetes [26]. Presently, the effects are unclear and controversial in both clinical and basic studies, with some investigators reporting that gliptins may reduce BP [27–29] and others reporting that these agents have no significant effects on BP [30, 31]. Ogawa et al. reported sitagliptin treatment affected SBP but not DBP in 17 patients with hypertension; however, in that research, the degree of decrease in HbA1c levels did not significantly correlate with the degree of decrease in SBP (r = 0.24) [28]. The results were, therefore, concordant with the present result in patients with mild diabetes but at high CV risk.

DPP-4 inhibition with sitagliptin increases the activity of GLP-1, which has been reported to decrease urinary salt uptake and increase urinary salt excretion [32]. In fact, 6 months of treatment with exenatide, a GLP-1 receptor agonist, was associated with a greater reduction in SBP compared with placebo or insulin in pooled data from six trials including 2171 patients [33]. The DURATION-2 study assessed the safety and efficacy of once weekly exenatide against maximum approved doses of sitagliptin, thiazolidinedione, or pioglitazone, and indicated that the reduction in SBP was significantly greater with exenatide once weekly than with sitagliptin in all patients after 26 weeks of treatment [34]. The reason for the variation in the long-term effects of gliptins on BP remains unknown; however, it might be dependent on the patient’s physiological state because DPP-4 metabolizes both prohypertensive and antihypertensive peptides [35]. Further clinical studies are necessary to elucidate the reasons for these differences. In the present study, HR significantly increased both in ECG analysis and ambulatory BP measurement. It might be associated with the reactive activation in the sympathetic nerve system or inhibition of the vagal nerve system to the BP decrease as suggested in the treatment of GLP-1 receptor agonists [36]. However, the ΔHR was not associated with ΔSBP or ΔDBP at 12 months in the present study, and the other mechanism might be suggested. The point of the long-term effects or CV outcomes needs to be clarified with caution [37].

The development of albuminuria is a key step in the progression of diabetic kidney disease, and worsening of albuminuria is a significant predictor of both progressive renal disease and CVD in patients with diabetes. The mechanisms underlying albuminuria are complex, and improved insulin sensitivity, weight loss, reduced blood glucose levels, lower BP, and suppressed inflammation might all contribute to a reduction in albuminuria. Several studies have reported a significant correlation between improvements of albuminuria and BP in patients with diabetes, hypertension, or nephropathy. Kawasaki et al. reported in a retrospective analysis that sitagliptin reduced albuminuria, and that this was independently associated with reductions in BP and eGFR in 247 patients with diabetes [38]. In the present study, although sitagliptin did not reduce albuminuria overall, the reduction of BP was modestly associated with a decrease in albuminuria that was independent of ΔHbA1c. Groop et al. reported that treatment with linagliptin for diabetic patients with renal dysfunction led to a significant reduction in albuminuria, independent of changes in SBP [39]. Although these findings may suggest direct renoprotective effects of DPP-4 inhibitors [40], such a blood-pressure-independent effect was not observed in the present study. eGFR was deteriorated during the 12-month sitagliptin treatment, which was positively associated with ΔSBP or ΔDBP in the present study. Kawasaki et al. reported the similar findings and discussed that it might be related to the decrease in the glomerular internal pressure and be beneficial for the renal tissue [38]. This mechanism resembles the decrease in eGFR observed through the action of renin–angiotensin system inhibitors in decreasing glomerular internal pressure and albuminuria. Further evidence is needed to confirm these renal effects, particularly in the long-term and explore the underlying mechanisms in patients with diabetes [41].

### Study limitations

First, this was an uncontrolled study and the effects of sitagliptin were only assessed by comparison of measurements at three independent time points. It might be possible that the significant changes in several measures were affected by factors other than sitagliptin treatment. However, all the patients were educated about the importance of diet and physical exercise, and most had stable medical treatment at least 1 month before the study inclusion. Second, the study assessments took place at 3 or 12 months, and may have been too short for the assessment of CV effects and outcomes. Therefore, the analysis may have been insufficient to provide conclusive evidence for long-term CV effects and outcomes associated with sitagliptin. Future studies must examine the BP lowering effects of sitagliptin and its direct influence on CV outcomes. Third, most of the participants had early-stage diabetes (69.3 % had an HbA1c <7.0 %) and higher age (73.4 % were aged >65 years), meaning that our results cannot be extrapolated to all patients with diabetes and high CV risk. Finally, our study design also led to the exclusion of some patients with severe chronic kidney disease or end-stage renal disease, which limits the applicability of the data in these settings.

## Conclusion

We conducted a prospective, multicenter, observational study of 205 patients with type 2 diabetes at high risk of CVD who were treated with sitagliptin for 12 months. The efficacy of sitagliptin for glycemic control was demonstrated by the higher baseline HbA1c level and the reduction of hs-CRP levels. Sitagliptin also showed BP lowering effects over the 12-month study period associated with the prevention of microalbuminuria progression. However, there were no beneficial effects on cardiac and endothelial function or on serum BNP, hs-TnT, and urinary 8-OHdG levels. We conclude that in Japanese patients with diabetes at high risk of CVD, sitagliptin had beneficial effects on BP and glycemic control over 12 months and it might offer long-term advantages in the management of diabetes with comorbid CVD.

## Additional files

10.1186/s12933-016-0371-z Comparison of changes in HbA1c (ΔHbA1c) at different time periods by multivariate prediction models.
10.1186/s12933-016-0371-z Changes in pro-inflammatory and cardiovascular biomarkers.
10.1186/s12933-016-0371-z Relationship of changes in blood pressure (ΔSBP or ΔDBP) with those in heart rate (ΔHR) or those in eGFR (ΔeGFR) for 12 months.

## Abbreviations

AU: albuminuria
BNP: B-type natriuretic peptide
BP: blood pressure
CV: cardiovascular
CVD: cardiovascular disease
Dct: deceleration time of the mitral E-wave velocity
DPP-4: dipeptidyl peptidase-4
e′: peak of myocardial early diastolic velocity
E/A ratio: ratio of peak mitral E-wave velocity to peak mitral A-wave velocity
ECG: electrocardiography
EF: ejection fraction
eGFR: estimated glomerular filtration rate
GIP: glucose-dependent insulinotropic polypeptide
GLP-1: glucagon-like peptide-1
HbA1c: hemoglobin A1c
HDL: high-density lipoprotein
HOMA-IR: homeostasis model assessment-insulin resistance
HR: heart rate
hs-CRP: high-sensitive C-reactive protein
hs-TnT: high-sensitive troponin T
LV: left ventricular
8-OHdG: 8-hydroxy-2′-deoxyguanosine
RHI: reactive hyperemia index
RH-PAT: reactive hyperemia-peripheral arterial tonometry
SD: standard deviation
UACR: urinary albumin-to-creatinine ratio
UCG: echocardiography
