# Supplementary material for: Cardiovascular efficacy of sitagliptin in patients with diabetes at high risk of cardiovascular disease: a 12-month follow-up
Source: Cardiovasc Diabetol. 2016 Mar 31;15:54. doi: 10.1186/s12933-016-0371-z (PMC4818390; doi:10.1186/s12933-016-0371-z)
Supplement: Supplementary file 1 — 10.1186/s12933-016-0371-z Comparison of changes in HbA1c (ΔHbA1c) at different time periods by multivariate prediction models. [file 12933_2016_371_MOESM1_ESM.docx]

**Table S1. Comparison of changes in HbA1c (ΔHbA1c) at different time periods by multivariate prediction models**

|  | ΔHbA1c 0–3 M  (R^2^ = 0.369) | | ΔHbA1c 3–12 M  (R^2^ = 0.232) | | ΔHbA1c 0–12 M  (R^2^ = 0.273) | |
| --- | --- | --- | --- | --- | --- | --- |
|  | **β** | **P value** | **β** | **P value** | **β** | **P value** |
| Age | −0.010 | 0.879 | −0.285 | <0.001 | −0.207 | 0.005 |
| Male | 0.009 | 0.889 | −0.041 | 0.591 | −0.045 | 0.526 |
| Concomitant drug | 0.070 | 0.261 | −0.204 | 0.007 | −0.078 | 0.277 |
| Pre-HbA1c level | −0.608 | <0.001 | −0.152 | 0.046 | −0.435 | <0.001 |
| BW change | −0.017 | 0.784 | 0.254 | <0.001 | 0.148 | 0.042 |

BW, body weight; HbA1c, glycated hemoglobin; M, months.
