# Supplementary material for: Cardiovascular efficacy of sitagliptin in patients with diabetes at high risk of cardiovascular disease: a 12-month follow-up
Source: Cardiovasc Diabetol. 2016 Mar 31;15:54. doi: 10.1186/s12933-016-0371-z (PMC4818390; doi:10.1186/s12933-016-0371-z)
Supplement: Supplementary file 2 — 10.1186/s12933-016-0371-z Changes in pro-inflammatory and cardiovascular biomarkers. [file 12933_2016_371_MOESM2_ESM.docx]

**Table S2. Changes in pro-inflammatory and cardiovascular biomarkers**

|  | **Baseline** | **3 months** | **12 months** | **P Value** |
| --- | --- | --- | --- | --- |
| **hs-CRP (mg/dL)** | 2227.5 ± 5739.2 | 1185.3 ± 2010.8 | 1993.2 ± 6530.5 | 0.0994 |
| **BNP (pg/mL)** | 63.5 ± 75.4 | 65.8 ± 81.9 | 63.0 ± 76.6 | 0.8098 |
| **hs-TnT (ng/mL)** | 0.0076 ± 0.0072 | 0.0084 ± 0.0093 | 0.0122 ± 0.0128 | 0.0012 |
| **Urine 8-OHdG (ng/mg/Cr)** | 11.47 ± 7.65 | 11.73 ± 12.6 | 13.97 ± 18.43 | 0.3758 |

Values are the mean ± SD. BNP, B-type natriuretic peptide; hs-CRP, high-sensitive C-reactive protein; Cr, creatinine; hs-TnT, high-sensitive troponin T; 8-OHdG, 8-hydroxy-2′-deoxyguanosine.
