# Supplementary material for: Cardiovascular efficacy of sitagliptin in patients with diabetes at high risk of cardiovascular disease: a 12-month follow-up
Source: Cardiovasc Diabetol. 2016 Mar 31;15:54. doi: 10.1186/s12933-016-0371-z (PMC4818390; doi:10.1186/s12933-016-0371-z)
Supplement: Supplementary file 3 — 10.1186/s12933-016-0371-z Relationship of changes in blood pressure (ΔSBP or ΔDBP) with those in heart rate (ΔHR) or those in eGFR (ΔeGFR) for 12 months. [file 12933_2016_371_MOESM3_ESM.docx]

**Figure S1. Relationship of changes in blood pressure (ΔSBP or ΔDBP) with those in heart rate (ΔHR) or those in eGFR (ΔeGFR) for 12 months**


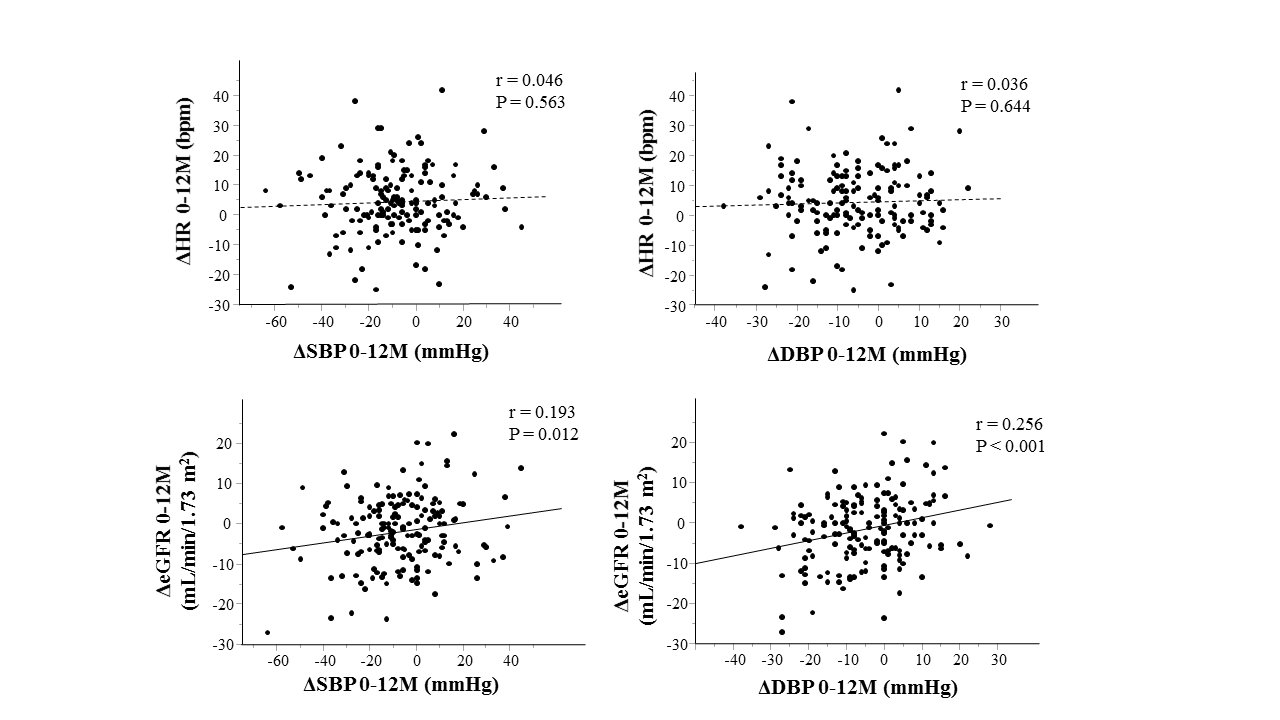


DBP, diastolic blood pressure; eGFR, estimated glomerular filtration rate; HR, heart rate; M, month; SBP, systolic blood pressure.
